# Overall Survival Prediction for Gliomas Using a Novel Compound Approach

**DOI:** 10.3389/fonc.2021.724191

**Published:** 2021-08-18

**Authors:** He Huang, Wenbo Zhang, Ying Fang, Jialing Hong, Shuaixi Su, Xiaobo Lai

**Affiliations:** School of Medical Technology and Information Engineering, Zhejiang Chinese Medical University, Hangzhou, China

**Keywords:** automatic segmentation, deep learning, gliomas, magnetic resonance imaging, overall survival prediction

## Abstract

As a highly malignant tumor, the incidence and mortality of glioma are not optimistic. Predicting the survival time of patients with glioma by extracting the feature information from gliomas is beneficial for doctors to develop more targeted treatments. Magnetic resonance imaging (MRI) is a way to quickly and clearly capture the details of brain tissue. However, manually segmenting brain tumors from MRI will cost doctors a lot of energy, and doctors can only vaguely estimate the survival time of glioma patients, which are not conducive to the formulation of treatment plans. Therefore, automatically segmenting brain tumors and accurately predicting survival time has important significance. In this article, we first propose the NLSE-VNet model, which integrates the Non-Local module and the Squeeze-and-Excitation module into V-Net to segment three brain tumor sub-regions in multimodal MRI. Then extract the intensity, texture, wavelet, shape and other radiological features from the tumor area, and use the CNN network to extract the deep features. The factor analysis method is used to reduce the dimensionality of features, and finally the dimensionality-reduced features and clinical features such as age and tumor grade are combined into the random forest regression model to predict survival. We evaluate the effect on the BraTS 2019 and BraTS 2020 datasets. The average Dice of brain tumor segmentation tasks up to 79% and the average RMSE of the survival predictive task is as low as 311.5. The results indicate that the method in this paper has great advantages in segmentation and survival prediction of gliomas.

## Introduction

Gliomas are the most common primary malignant brain tumors (1). The incidence of primary intracranial tumors is 23 per 10 million, and gliomas account for about 60% (2). According to the degree of malignancy, the World Health Organization divides gliomas into low-grade gliomas (LGG) and high-grade gliomas (HGG). Different grades of gliomas have different levels of invasion and variable prognosis, which seriously threatens human health. The best treatment is complete surgical resection, however, due to the unresectable nature of normal brain tissue and the widespread infiltration of malignant tumors into the brain, complete resection surgery is extremely difficult (3). Therefore, early detection of tumors and targeted treatment play a vital role in prolonging the survival time of patients.

Magnetic resonance imaging (MRI) is one of the most common methods to obtain brain tumor images, it usually has multiple modes, namely fluid attenuation inversion recovery (Flair), T1 weighted (T1), T1 weighted contrast enhancement (T1-CE) and T2 weighted (T2) (4). Medical image analysis usually needs to segment the tumor first, but the manual segmentation method often depends on the doctor’s experience, knowledge and emotions, and the efficiency is low. Moreover, doctors can only observe image slices in a fixed manner, and cannot directly extract feature information in the segmentation results to quantify the diagnosis results, which makes the diagnosis have subjective experience. The development of machine learning and deep learning can change this situation. Through image processing technology, the computer can accurately and quantitatively analyze the tumor area, and make efficient and accurate survival prediction for patients with brain tumors, so as to formulate personalized diagnosis and treatment plans for patients.

Up to now, the overall survival prediction of glioma patients with multimodal MRI has received widespread attention (5). It is observed that most survival prediction models proposed in the literature are based on radiomics (6). Although radiological features extracted from images can be used to predict tumor grade or molecular biomarkers (7, 8), it is difficult to accurately predict survival time without considering other factors such as age. In this article, we propose an automatic prediction method by combining radioactive features, deep features and clinical features to provide accurate survival predictions for patients with glioma. The major contributions of our work are four folds that can be summarized as follows:

- We improved the V-Net and proposed NLSE-VNet to segment brain tumors. The innovation of the NLSE-VNet model lies in porting the Non-Local (NL) module and Squeeze-and-Excitation (SE) module to the V-Net network structure. These attention models can enhance feature extraction capabilities. We have designed multiple ablation experiments to prove that NLSE-VNet can greatly improve the accuracy of brain tumor segmentation.
- We extracted various types of radiological features such as intensity, texture, wavelet, etc., and designed a CNN network to extract deep features. After cross-validation and a large number of comparative experiments, it is proved that when radiological features, deep features and clinical features are combined, the effect of predicting survival is the best.
- We perform three-dimensional reconstruction of the segmentation results to provide clinicians with a visual display. At the same time, in order to improve the interpretability of feature dimensionality reduction, we draw feature heat maps to show how the model finds meaningful features, which provides a reliable basis for the clinical application of survival prediction.

The remainder of this paper is organized as follows. The second section describes the previous research on MRI segmentation and survival prediction of glioma. The third part introduces the method proposed in this paper in detail, including data preprocessing, model description and parameter setting. *Dataset and Experiments* introduces the data set, evaluation indicators and experimental configuration. Then, in *Experimental Results*, the experimental results are presented and discussed and analyzed. Finally, conclusions are drawn in *Conclusion*.

## Related Works

### Brain Tumor Segmentation

The segmentation of the tumor area is a prerequisite for prediction. At present, brain tumor segmentation has received extensive attention and in-depth research, and various excellent models have been proposed for segmenting brain tumors. Zhou et al. improved the model cascade strategy and proposed a single multi-task network (OM-Net) (9), which can solve the problem of category imbalance. OM-Net integrates the separated segmentation tasks into a deep model, which is shared by Parameters to learn joint features and task-specific parameters to learn discriminative features. At the same time, they designed a CGA attention module that can adaptively recalibrate the characteristics of the channel direction. S. Pereira et al. designed a deep-level architecture based on a convolutional neural network using a small 3×3 kernel (1). The model has a positive effect on overfitting when the network weight is less. In the training process, the number of LGG classes is increased by rotating training patches and using HGG samples, and the number of training patches is artificially increased. Chen et al. established a model based on 3D convolutional neural network to segment brain tumors (10). The model obtains multi-scale context information by extracting the features of two scales of the receptive domain, and they use hierarchical segmentation to segment different lesion areas such as necrotic and non-enhanced tumors, peritumoral edema, and enhanced tumors, using densely connected The convolution block further improves performance. Sun et al. proposed an anatomical attention-guided deep learning framework for segmenting brain tumors (11). It contains two sub-networks, one is the segmentation sub-network, and the other is the anatomical attention sub-network, so as to combine the anatomical structure information of the brain with The feature information in the segmentation process is combined to improve performance. Lachinov et al. proposed an automatic segmentation algorithm for brain tumors based on deep cascades (12). Their team modified the 3D U-Net architecture and designed 4 down-sampling paths to extract the features of the four modalities of brain tumors. The model can effectively process input images on multiple scales at the same time and extract features of specific scales.

### Overall Survival Prediction

Overall survival prediction of cancer patients has also been a hot research topic in recent years. Sun et al. extracted 4,524 radiomic features from the segmented area of the tumor, then used decision trees and cross-validation to select effective features, and finally trained a random forest model to predict the survival of patients (13). Shboul et al. extracted about 31,000 features from the tumor area, representing texture, volume, area, and Euler features. Then they performed recursive feature selection on Euler features separately, and finally used XGBoost to predict survival (14). Baid et al. calculated the first-order statistics, shape features, gray-level co-occurrence matrix and gray-level run length matrix for a total of 679 features (15). The radiation group variables with Spearman correlation coefficients of 0.95 and above that are nearly completely correlated were excluded, and the features were reduced to 56 dimensions, using multilayer perceptrons to train the neural network to predict the number of survival days. Kim et al. extracted a total of 6472 radiological features from multi-mode MR images, applied the Least Absolute Shrinkage and Selection Operator (LASSO) to the training data set to select significant non-zero coefficient radiation features, and constructed a radiation group using a generalized linear model to predict the survival period with a scientific model (16). Weninger et al. used the volume characteristics of all regions of interest, the distance from the brain to the center of mass of the tumor, and input the age into the linear regression model when predicting survival (17). They found that using only the “age” feature to train the regression model achieved higher accuracy on the test set. Banerjee et al. extracted two types of radiological features, namely “semantic” and “agnostic” (18). The former includes attributes such as size, shape, and location, while the latter uses histograms, textures, and other quantitative descriptions to capture the heterogeneity of lesions. A total of 83 features were extracted as the input of the multi-layer perceptron to predict the number of survival days. Wang et al. used internal radiomics analysis software to extract 43 unique quantitative features in 4 categories, selected features with high r values in related tests, used support vector regression SVR to predict OS, and used leave-one-out cross-validation (LOOCV) (19).

### Our Work

Although computer technology has made significant progress in the field of brain tumor segmentation and survival prediction, there are still some challenges that prevent this fully automated technology from being well applied in clinical practice. First, the size, location, and shape of brain tumors vary from patient to patient (20). Secondly, the lesion area in MRI is very small in most cases, which leads to the voxel imbalance between the lesion area and the background area (10). The above challenges all increase the difficulty of segmentation. Finally, the information obtained from other data sources, such as genes and age, is usually not used when extracting radiomic features, which further limits the ability to distinguish predictions (21). Our goal is to innovate existing methods to improve the accuracy of segmentation and prediction on the basis of predecessors.

In this article, we first propose a brain tumor segmentation model NLSE-VNet, which is an improvement on the V-Net network structure. We transplant the Squeeze-and-Excitation (SE) module to the front of each down/up sampling layer of V-Net (22). It can clarify the interdependence between channels, and at the same time, the Non-Local (NL) modules are transplanted after the last encoder block in the network to capture long-term dependencies (23). The comparative experiment proves the validity of the model. Secondly, we use the Pyradiomics toolkit to extract radiological features such as intensity, texture, and filtering from the original and derived images, and designed a CNN network to extract deep features. Since most of the extracted features are redundant, we consider reducing the dimensionality of the features. After several experiments, the factor analysis method (FA) was used to reduce the feature dimension. Finally, a random forest model was constructed to predict the survival time by using the features, age, and tumor grade after dimensionality reduction. We have implemented a fully automatic method from brain tumor segmentation to survival prediction. The experimental results show that our proposed method has great potential in clinical application.

## Methods

**Figure 1:**
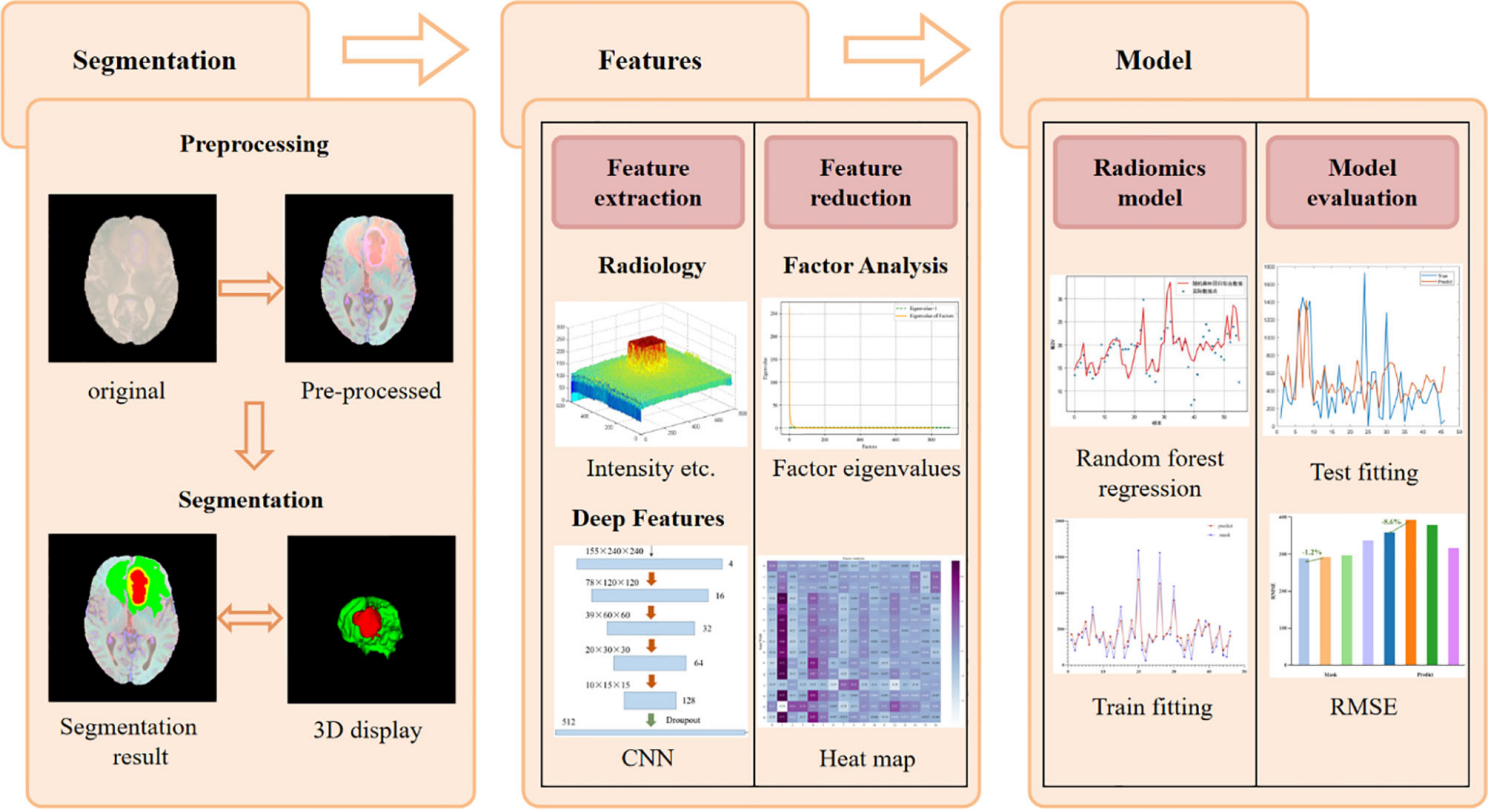
Flow chart of brain tumor segmentation and overall survival prediction.

The task of this article is to automatically segment brain tumors and predict the survival time of glioma patients. All the workflow of this project is shown in **Figure 1**, which is divided into five parts: brain tumor segmentation, feature extraction, feature dimensionality reduction, radiology model selection and model evaluation.

### Glioma Segmentation Model

**Figure 2:**
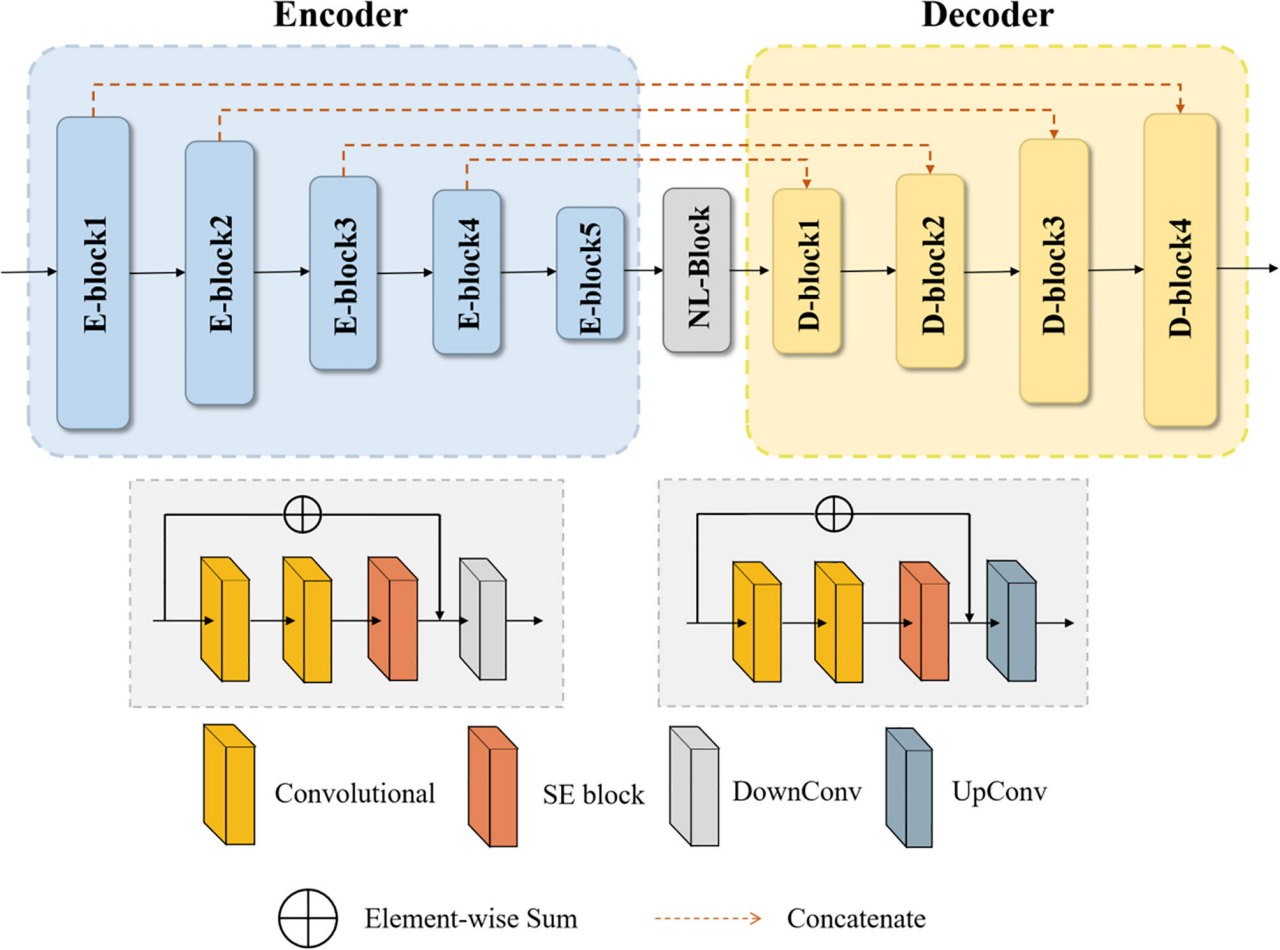
Schematic diagram of the NLSE-VNet for the glioma segmentation.

The basic idea of the NLSE-VNet model is to achieve feature information extraction of images of different resolutions through alternating convolutional layers and down-sampling layers, and then use the features extracted by the up-sampling layer joint encoder to gradually achieve resolution restoration. The SE module is placed in front of the down-sampling layer and the up-sampling layer, and the NL module is placed behind the last down-sampling layer. These attention modules bring a significant improvement in segmentation accuracy while slightly increasing the computational cost. Its network structure is shown as in **Figure 2**. We will introduce from four aspects: preprocessing process, network structure, loss function and model training.

#### Data Preprocessing and Data Augmentation

**Figure 3:**
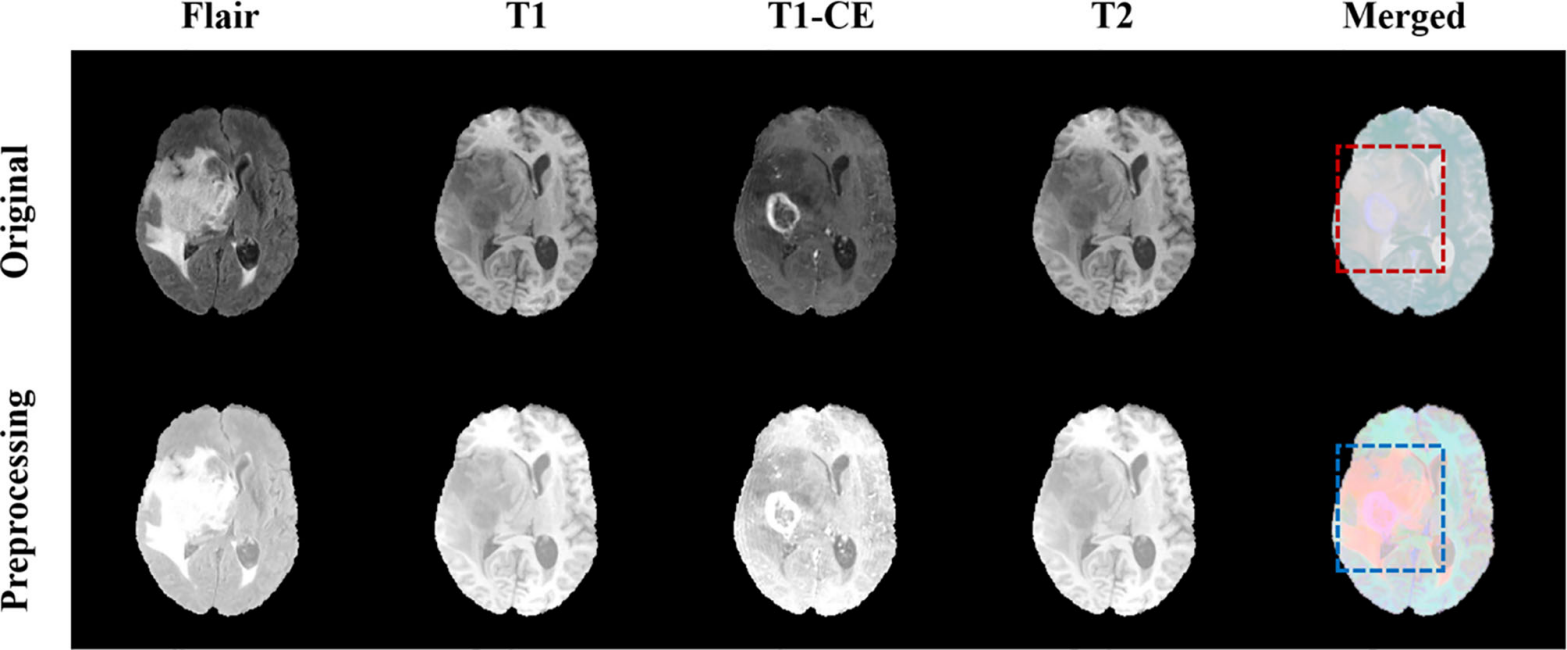
Comparison of MRI before and after pretreatment. The first line is the original image, the second line is the preprocessed image. The first four columns are the images of the four modalities Flair, T1, T1-CE, and T2, and the last column is the merged image.

In addition, we use the Z-score method to standardize the images of the four modalities, and then merge the standardized images as input to the model. Z-score is the image minus the mean divided by the standard deviation; its mathematical formula is as follows:

(1) $$Z=\frac{X-\bar{X}}{s}$$

where *Z* represents the normalized image, *X* represents the original image, $\bar{X}$ represents the pixel average, and *s* represents the pixel standard deviation. **Figure 3** shows the comparison images before and after preprocessing. The first four columns are the comparison before and after the pre-processing of the four modes, Flair, T1, T1-CE, and T2, respectively, and the last column is the comparison after combining the four modes. It can be seen from **Figure 3** that after preprocessing, the contrast of the tumor part is significantly enhanced compared to normal tissue, which facilitates image segmentation.

The data enhancement method we use is patch. Combine the preprocessed MRI of the four modalities to generate a three-dimensional image with four channels, and then divide the original image and the mask into multiple blocks. One case will generate 175 pictures with a size of 128×128×64×4. This makes it possible to process only one patch instead of the entire image, thereby better detecting edge features.

#### Network Architecture

On the basis of the VNet network, we introduced the Squeeze-and-Excitation (SE) module and the Non-Local (NL) module. The 3D SE module uses feature recalibration to explicitly model the interdependence between feature channels, that is, the importance of each feature channel is automatically obtained through learning. The specific process of the SE block module is as follows, the input is $X\in {\mathbb{R}}^{Z\times H\times W\times C}$, where Z is the depth, H is the height, W is the width, and C is the number of channels. Then the input image will undergo a global average pooling, called squeeze operation, the formula is as (2):

(2) $$z_{c}=F_{sq}(x_{c})=\frac{1}{Z\times H\times W}\sum_{i=1}^{Z}\sum_{j=1}^{H}\sum_{k=1}^{W}\;x_{c}(i,j,k)$$

**Figure 4:**
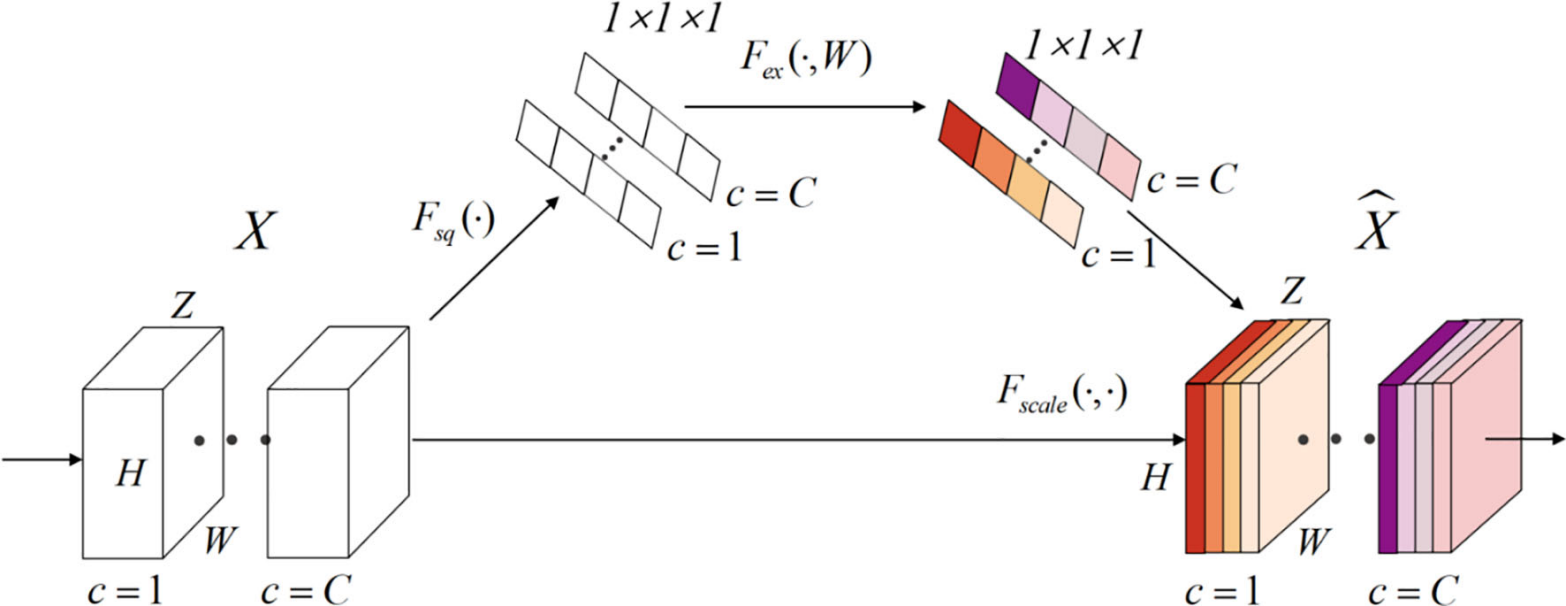
Structure of Squeeze-and-Excitation module (SE).

The excitation operation is used to utilize the information aggregated in the squeeze operation, and the excitation part is composed of two fully connected layers. The first full connection compresses the *C* channel into a *C/r* channel to reduce the amount of calculation, and the second full connection returns to the *C* channel. *r* is the compression ratio. The calculation of the excitation part is shown in (3):

(3) $$s=F_{ex}(z,\;W)=\sigma \;(g\;(z,\;W))=\sigma \;(W_{2}\;\delta \;(W_{1}\;z))$$

where *δ* represents the ReLU activation function, $W_{1}\in {\mathbb{R}}^{\frac{C}{r}\times C}\text{ and }W_{2}\in {\mathbb{R}}^{C\times \frac{C}{r}}$. Finally, by re-calibrating *X* by activatings, the final output of the block can be obtained as (4):

(4) $${\hat{x}}_{c}=F_{scale}(x_{c},\;s_{c})=s_{c}x_{c}$$

where $\hat{X}=[{\hat{x}}_{1},\;{\hat{x}}_{2},\text{...},{\hat{x}}_{C}]$ and *F_scale_*(*x_c_*, *s_c_*) refers to channel-wise multiplication. The structure of SE module is shown as in **Figure 4**.

**Figure 5:**
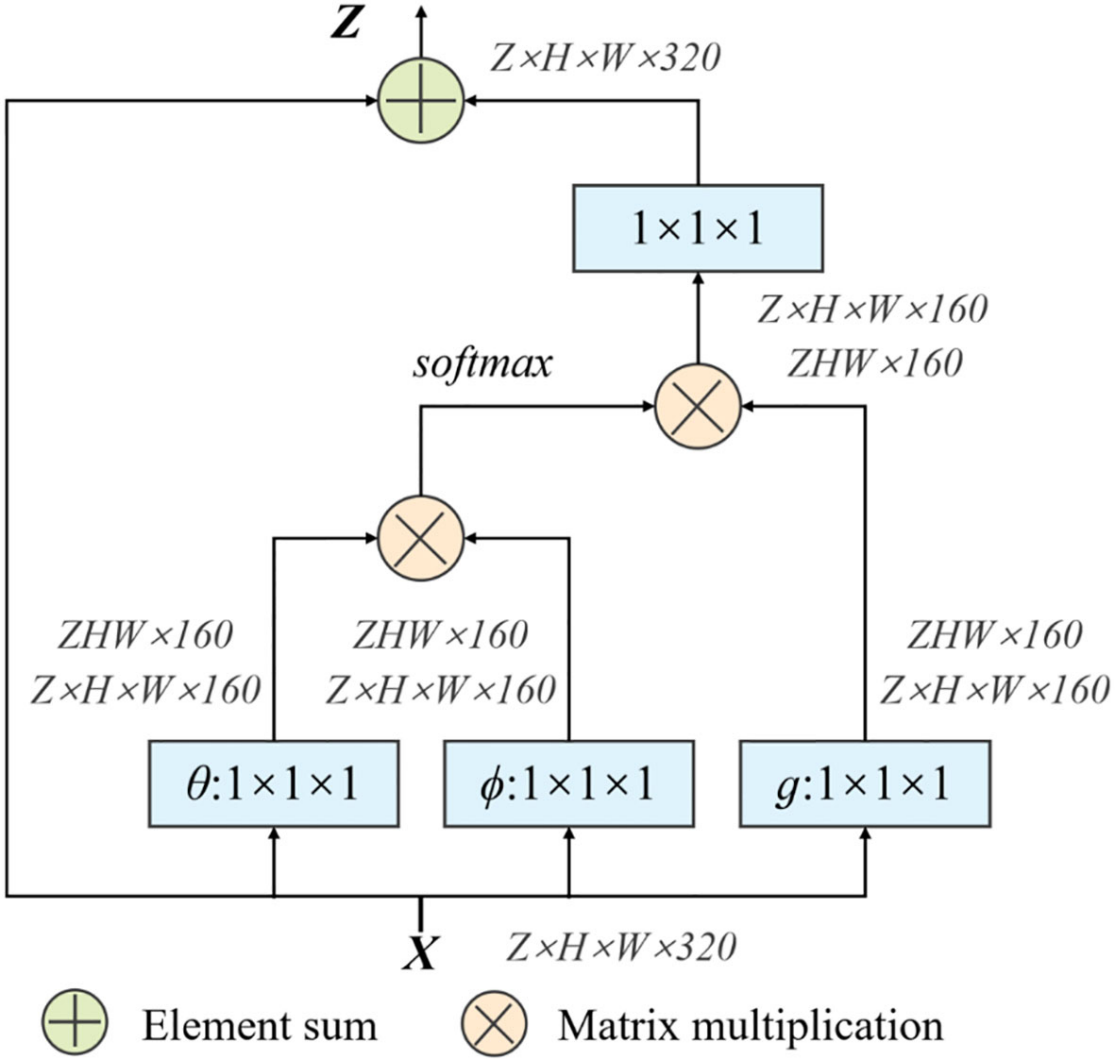
Structure of Non-Local module (NL).

At the same time, in order to quickly capture remote dependencies and improve computational efficiency, we have also introduced a 3D Non-Local module, which is integrated after the model downsampling stage. Its structure is shown in **Figure 5**.

First of all, the network input *X* = (*Z*, *H*, *W*, 320), after the weight matrix *W_θ_*, *W_ϕ_* transform respectively, use the 1×1×1 size convolution kernel to perform convolution operation to reduce the number of channels. Then reshape the two outputs to *ZHW*×160, then perform matrix multiplication and perform softmax processing. At the same time, the weight matrix *W_g_* is performed on *X* again, and the convolution operation of 1×1×1 is used. Perform a matrix multiplication of this result with the output result of softmax in the previous step. Finally, the output channel is restored through a 1×1×1 convolution operation to ensure that the input and output sizes are exactly the same.

#### Loss Function

Considering that the tumor area we want to segment only occupies a small part of the entire scanning area, and the proportion of foreground and background area is extremely unbalanced, we choose Categorical Dice as the loss function of the model to prevent the prediction from being strongly biased towards the background area that we are not interested in. The Categorical Dice is an improvement based on the Generalized Dice loss function (GDL) proposed by Sudre C.H. et al. (24). The generalized dice loss function has been proved to be able to effectively solve the problem of brain tumor imbalance. Its calculation formula is:

(5) $$\text{GDL}=1-2\frac{\sum_{l=1}^{2}\omega_{l}\sum_{i=1}^{N}p_{\ln}g_{\ln}}{\sum_{l=1}^{2}\omega_{l}\sum_{i=1}^{N}p_{\ln}+g_{\ln}}$$

The weight is defined as $\omega_{l}=1/{\left(\sum_{n=1}^{N}g_{\ln}\right)}^{2}$. Where N represents all voxels, *l* represents the number of categories, *p* represents the predicted voxel, *g* represents the real voxel. And we assign the weights of different categories. We set the weight of the background area to 0.1, and the weight of the gangrene, edema and enhanced tumor area to 1.0, and the value of *ω* is [0.1, 1.0, 1.0, 1.0]. Through this weight distribution, the problem that the weight assigned to the background area tends to 0 when there are too many voxels is avoided. The calculation formula is shown in (6):

(6) $$\text{Loss}=-2\frac{\sum_{l=1}^{4}\omega_{l}\sum_{i=1}^{N}p_{\ln}g_{\ln}}{\sum_{l=1}^{4}\omega_{l}\sum_{i=1}^{N}p_{\ln}+g_{\ln}}$$

#### Model Training

The realization of the segmentation task is based on tensorflow 1.13.1. In addition, we used the Adam optimizer to train the model. The entire network was trained with a total of 500,000 steps and the training set was traversed 10 times. After each traversal of the training set, the order of the data will be randomly shuffled to enhance the robustness of training. The initial learning rate is set to 0.0001, which is reduced to half of the original learning rate after traversing the training set twice. Finally, Mean Dice is used as the evaluation index of training, and the loss value and accuracy index are output every ten steps to realize effective supervision of model training. At the same time, the parameter model is saved every 1000 steps.

The experimental environment is run on TensorFlow. The runtime platform processor is Intel (R) Xeon (R) Silver 4210 CPU @2.20GHz 2.20GHz 128GB RAM, Nvidia Titan RTX, 64-bit Windows10.The development software platform is PyCharm with Python 3.6.

### Overall Survival Prediction

**Figure 6:**
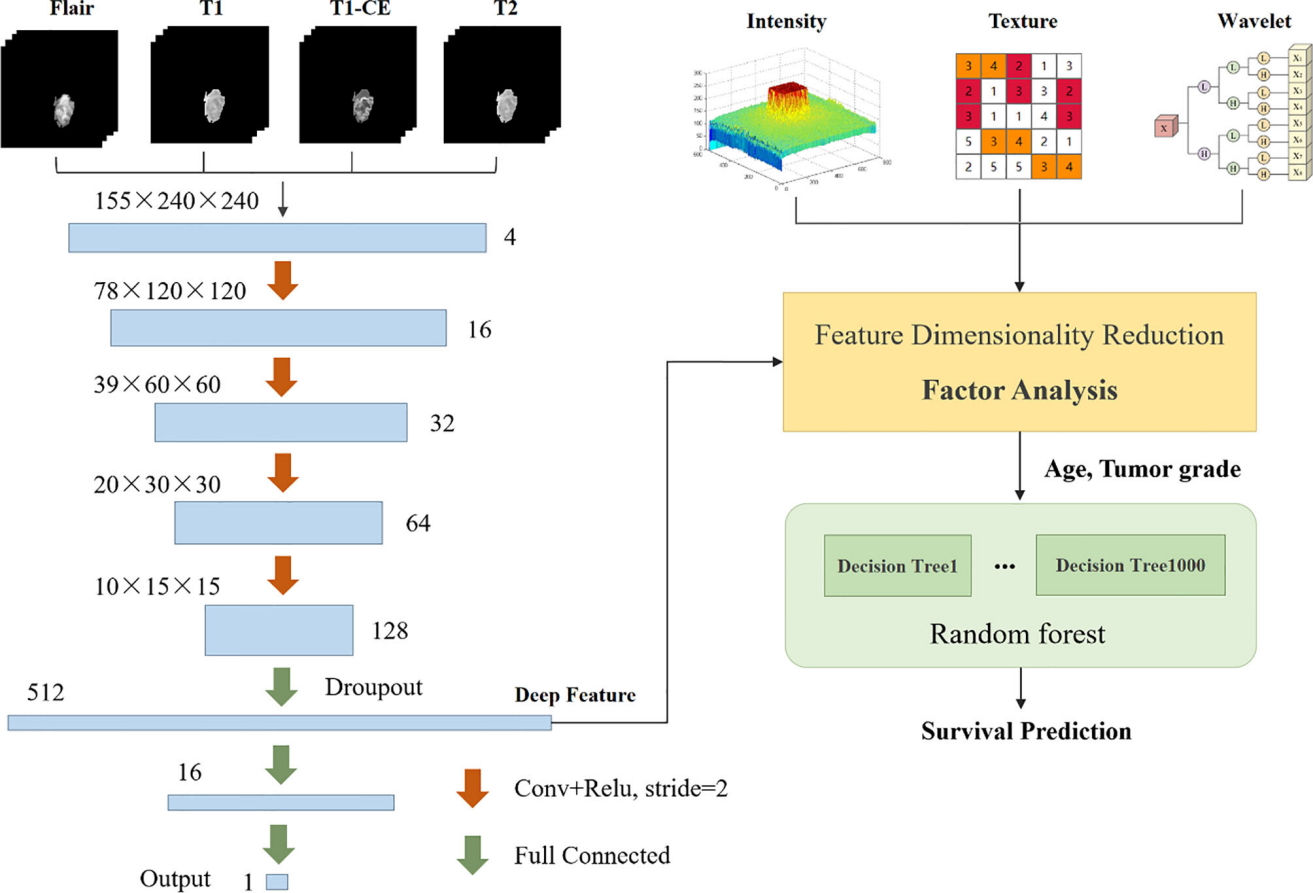
Summary of prediction methods for the survival period of patients with brain tumors.

In the survival prediction task, we extract radiological features such as intensity, texture, and filtering, and then build a CNN network to extract deep features. This network can also be used to predict survival. Subsequently, factor analysis is used to reduce the dimensions of the above two types of features to remove redundant features. Finally, the dimensionality reduction features combined with clinical factors such as age and tumor grade are input into the random forest regression model for survival prediction. The entire flow chart is shown in **Figure 6**.

#### Radiological Feature Extraction

**Figure 7:**
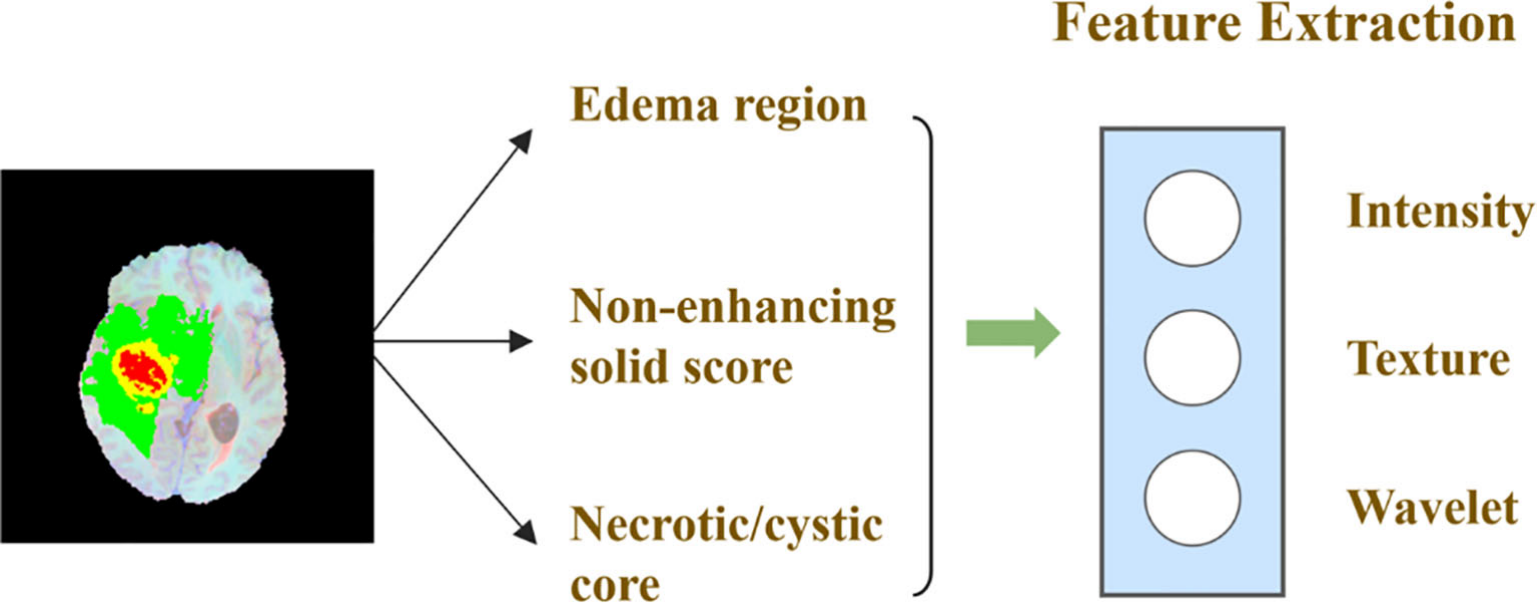
Schematic diagram of image feature extraction.

Based on the segmentation results, we use the Pyradiomics toolbox to extract the radiomic features of edema, non-enhanced verification, and necrosis/cystic nucleus areas. We mainly extract three types of features: intensity, texture, and wavelet, as shown in **Figure 7**. Pyradiomics is an open source Python software package that can extract the features of Radiomics from medical images (25).

Then, we further subdivide the extracted radiological features into 7 categories. The first-order statistical features reflect the voxel intensity distribution in the image area defined by the mask. The shape is based on 3D shape features, including a series of tumor shape features, such as sphericity, circumference ratio, spindle length, and elongation. The Gray Level Co-occurrence Matrix (GLCM) defines information about correlation, energy, contrast, deficiency, variance, probability, entropy, sum of squares, etc. In addition, we extracted 16 features of the gray run length matrix (GLRLM), 16 features of the gray size band matrix (GLSZM), 14 features of the gray dependency matrix (GLDM) and neighbor gray tone difference Five features of the matrix (NGTDM). **Table 1** shows the detailed information of feature categories.

**Table 1:** Types and contents of extracted features.

Feature Category	Name of the features
First Order Statistic	Energy, Total Energy, Entropy, Minimum, 10th percentile, 90th percentile, Maximum, Mean, Median, Interquartile Range, Range, Mean Absolute Deviation, Robust Mean Absolute Deviation, Root Mean Squared, Standard Deviation, Skewness, Kurtosis, Variance, Uniformity.
Shape	Mesh Volume, Voxel Volume, Surface Area, Surface Area to Volume ratio, Sphericity, Compactness 1, Compactness 2, Spherical Disproportion, Maximum 3D diameter, Maximum 2D diameter (Slice), Maximum 2D diameter (Column), Maximum 2D diameter (Row), Major Axis Length, Minor Axis Length, Least Axis Length, Elongation, Flatness.
GLCM	Autocorrelation, Joint Average, Cluster Prominence, Cluster Shade, Cluster Tendency, Contrast, Correlation, Difference Average, Difference Entropy, Difference Variance, Joint Energy, Joint Entropy, Informational Measure of Correlation, Informational Measure of Correlation, Inverse Difference Moment, Maximal Correlation Coefficient, Inverse Difference Moment Normalized, Inverse Difference, Inverse Difference Normalized, Inverse Variance, Maximum Probability, Sum Average, Sum Entropy, Sum of Squares.
GLRLM	Short Run Emphasis, Long Run Emphasis, Gray Level Non Uniformity, Gray Level Non-Uniformity Normalized, Run Length Non-Uniformity, Run Length Non-Uniformity Normalized, Run Percentage, Gray Level Variance, Run Variance, Run Entropy, Low Gray Level Run Emphasis, High Gray Level Run Emphasis, Short Run Low Gray Level Emphasis, Short Run High Gray Level Emphasis, Long Run Low Gray Level Emphasis, Long Run High Gray Level Emphasis.
GLSZM	Small Area Emphasis, Large Area Emphasis, Gray Level Non Uniformity, Gray Level Non-Uniformity Normalized, Size Zone Non-Uniformity, Size-Zone Non-Uniformity Normalized, Zone Percentage, Gray Level Variance, Zone Variance, Zone Entropy, Low Gray Level Zone Emphasis, High Gray Level Zone Emphasis, Small Area Low Gray Level Emphasis, Small Area High Gray Level Emphasis, Large Area Low Gray Level Emphasis, Large Area High Gray Level Emphasis.
GLDM	Small Dependence Emphasis, Large Dependence Emphasis, Gray Level Non-Uniformity, Gray Level Non-Uniformity Normalized, Dependence Non-Uniformity, Dependence Non-Uniformity Normalized, Gray Level Variance, Dependence Variance, Dependence Entropy, Dependence Percentage, Low Gray Level Emphasis, High Gray Level Emphasis, Small Dependence Low Gray Level Emphasis, Small Dependence High Gray Level Emphasis, Large Dependence Low Gray Level Emphasis, Large Dependence High Gray Level Emphasis.
NGTDM	Coarseness, Contrast, Busyness, Complexity, Strength.

We not only extract features from the original image, but also extract the same features from the image after wavelet decomposition. Wavelet decomposition can segment the image into multiple levels of detail components. In the end, we extracted a total of 2500 radiological features.

Deep learning has been used to predict the survival of patients with brain tumors. We tried to build a CNN network for the survival regression task, as shown in **Figure 6**. With the help of the segmentation result, we set the MR sequence to retain only part of the tumor information, and set other pixels to 0 as input. The CNN network consists of four convolutions with a step size of 2 and three fully connected layers. The last fully connected layers are directly used to predict survival time. The model can either extract deep features or directly use neural networks to predict survival days. In the end, we extracted 512 deep features. Since the CNN network can also learn the shape, texture of the brain tumor, these features are the same as part of the radiology features, we confirmed this in *Overall Survival Prediction Results*. So we believe that the deep features and radiological features should perform feature reduced together, which helps filter out repeat features.

#### Feature Dimensionality Reduction

**Figure 8:**
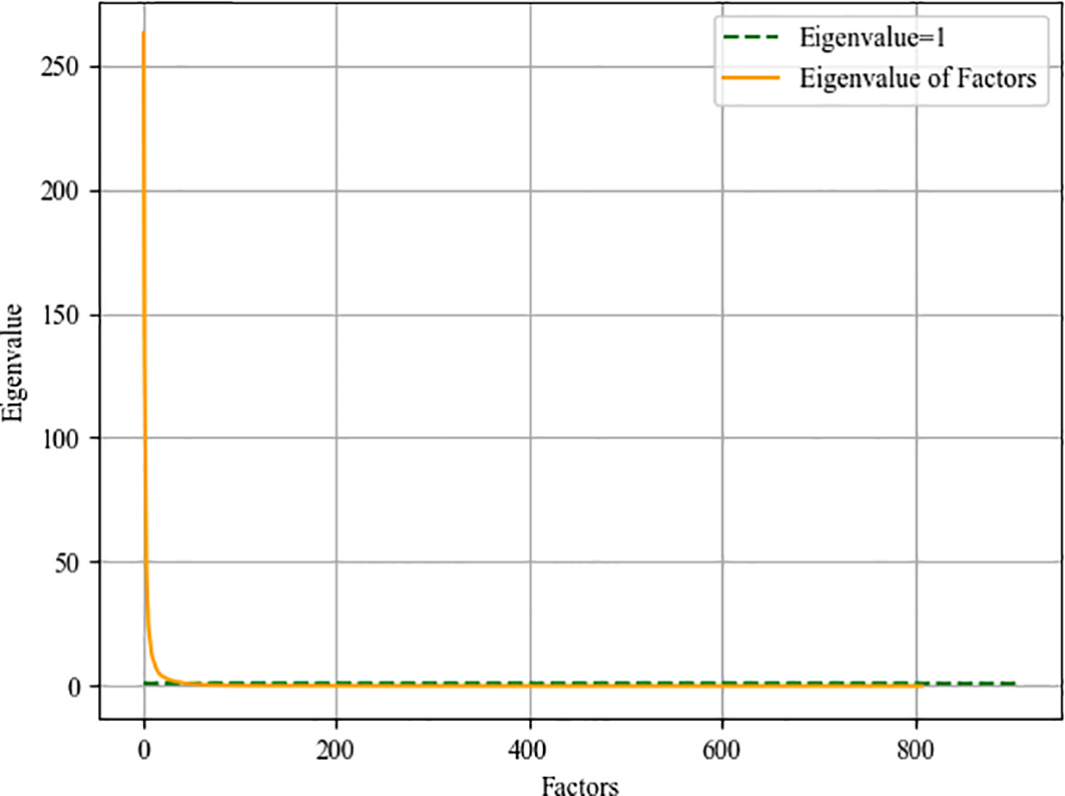
Eigenvalue curve of factor.

Some of the features we extract are redundant or have nothing to do with survival prediction, which will increase the degree of model overfitting, here we use factor analysis to filter features. The core of factor analysis is to analyze a series of features and extract common factors to achieve the purpose of reducing feature dimensions. First, the feature is used as a factor, and the feature value of the factor is calculated. The factor with the eigenvalues greater than 1 can be used as the subsequent feature dimensionality reduction. **Figure 8** shows the feature values of all the factors after sorting.

**Figure 9:**
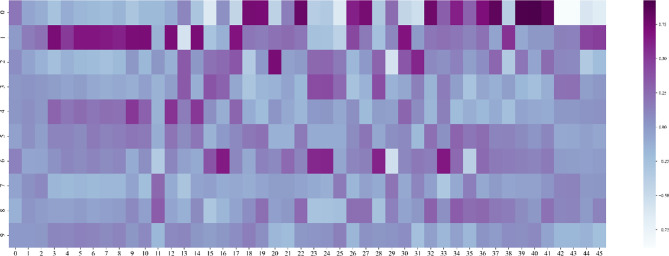
Heat map of feature dimensionality reduction.

Then we need to determine the dimensionality of the feature after dimensionality reduction, that is, recursively select within the range of factors whose feature value is greater than 1. Traverse the number of factors, reduce the feature to this dimension every two steps, enter the random forest model for training, and save the feature dimension with the smallest root mean square error. We draw a heat map of the process of feature dimensionality reduction, as shown in **Figure 9**. The abscissa represents the features before dimensionality reduction, and the ordinate represents the features after dimensionality reduction (Use numbers to represent feature names). It can be seen the degree of correlation between the original feature and the feature after dimensionality reduction.

#### Random Forest Model

After obtaining the effective feature set, we choose the random forest regression model to predict the survival period. Random forest algorithm is an ensemble technology that combines multiple decision trees, it usually has better generalization capabilities and not sensitive to multiple collinearity. Random Forest randomly selects k new self-service sample sets from the original training data set each time by applying the bootstrap method with replacement to construct k decision trees, and the unselected sample sets are used to estimate the generalization error of the model. Secondly, randomly extract *m* features at the node of each tree (*m* is less than the total number of features), and select the best split point to split by calculating the amount of information entropy in each feature. The calculation formula of information entropy is:

(7) $$S=-\sum_{i=1}^{n}P_{i}\log_{2}P_{i}$$

where *P_i_* represents the probability of occurrence of the i-th situation. Finally, the generated trees are formed into a random forest, and the random forest is used to classify the new data. The classification result is determined by the number of votes of the tree classifier.

We use 1000 basic decision tree regressors. In order to ensure the robustness of model training, we will cross-validate the model 100 times and randomly divide the data into a training set and a test set each time. The training set data accounted for 0.9 of the total, and the test set data accounted for 0.1 of the total. We make sure to use different data combinations for training and testing every time. Finally, the average of 100 cross-validations is used as the final error loss.

## Dataset and Experiments

### Datasets

The dataset we use comes from the 2019 and 2020 Brain Tumor Segmentation Challenge. The data set contains two types of tumors, namely high-grade glioblastoma (HGG) and low-grade glioblastoma (LGG). The MRI of each sample in the tumor segmentation task data contains four modalities: fluid attenuation inversion recovery (FLAIR), T1 weighting (T1), T1-weighted contrast-enhanced (T1-CE), and T2 weighting (T2). The tumor sub-regions are edema, non-enhanced verification, necrosis/cystic nucleus and the entire tumor. We need to segment the enhancing tumor (ET), whole tumor (WT), and tumor core (TC) formed by nesting these sub-regions. The organizer provided 335 training samples and 125 validation samples without masks in BraTS 2019. In the survival prediction task, 209 samples containing age, tumor grade (HGG and LGG) and survival time defined in days are provided. Also in BraTS 2020, the training set and validation set sizes are 369 and 125, and 236 survival samples are provided. We divide the survival data into training set and test set by 9:1 respectively for cross-validation.

### Evaluation Metrics

For the evaluation indicators of the segmentation model, we follow the 4 indicators used in the Brain Tumor Segmentation Challenge, namely Dice, Sensitivity, Specificity and Hausdorff distance. Dice is the overall evaluation standard, and its formula is defined as:

(8) $$\text{Dice}=\frac{2\text{TP}}{\text{FP}+2\text{TP}+\text{FN}}$$

where FN, TP and FP represent the number of false negative, true positive and false positive voxels respectively. Sensitivity represents the sensitivity of the model to voxels of the segmented region, and is used to measure the accuracy of segmenting the target region and is defined as:

(9) $$Sensitivity=\frac{\text{TP}}{\text{TP}+\text{FN}}$$

Specificity represents the ability of the model to correctly predict the background and is defined as:

(10) $$\text{Specificity}=\frac{\text{TN}}{\text{FP}+\text{TN}}$$

where TN represents the number of true negative. In addition, Hausdorff95 is a measure of Hausdorff distance. It is more sensitive to the segmentation boundary. The smaller the value, the closer the prediction is to the true value.

(11) $$\text{Hausdorff}\_95(X,Y)=\text{max}\{\underset{x\in X}{\text{max}}\underset{y\in Y}{\text{mind}}(x,y),\underset{y\in Y}{\max}\underset{x\in X}{\min d}(x,y)\}$$

where *X* is the volume of the mask, *Y* is the volume predicted by the model, and d(), represents the distance from *X* to *Y*.

In addition, for the survival prediction task, we choose MSE, MAE and RMSE as the evaluation indicators of the model. They are all used to measure the deviation between the predicted value and the true value. The calculation formula is as follows:

(12) $$\text{MSE}=\frac{1}{n}\sum_{i=1}^{n}{\left(X_{obs,i}-X_{pre,i}\right)}^{2}$$

(13) $$\text{MAE}=\frac{1}{n}\sum_{i=1}^{n}\left|X_{obs,i}-X_{pre,i}\right|$$

(14) $$\text{RMSE}=\sqrt{\frac{\sum_{i=1}^{n}{\left(X_{obs,i}-X_{pre,i}\right)}^{2}}{n}}$$

where *i* represents the patient, *n* represents the total number of patients, *X_obs_* represents the true survival period of the patient, and *X_pre_* represents the survival period predicted by the model.

## Experimental Results

### Segmentation Result

The brain tumor segmentation method proposed in the article is evaluated experimentally on the BraTS 2019, 2020 dataset. The data has a multi-modal imaging protocol: Flair, T1, T1-CE, T2. The mask is manually segmented by experts, including three nested sub-regions: enhanced tumor (ET), whole tumor (WT) and tumor core (TC) (5, 26). We train on the 3D volume of brain tumors, using dice coefficients, sensitivity, specificity and Hausdorff95 distance to evaluate the performance of the model. **Table 2** shows the average performance of the model on the validation set, and it can be seen that the model has achieved good segmentation results.

**Table 2:** Segmentation results of different models for BraTS 2019 and BraTS 2020.

	Dice			Sensitivity			Specificity			Hausdorff95		
	ET	WT	TC	ET	WT	TC	ET	WT	TC	ET	WT	TC
**BraTS2019**	0.70	0.87	0.74	0.73	0.87	0.70	0.99	0.99	0.99	5.7	7.4	9.8
**BraTS2020**	0.73	0.88	0.79	0.77	0.89	0.82	0.99	0.99	0.99	36.9	6.5	10.0

At the same time, we conducted ablation experiments on BraTS 2020 dataset, removing the SE module, NL module and the V-Net model without any attention module. Since CNN is the basis for the automatic segmentation of brain tumors, we also designed a CNN network to segment brain tumors as a control group. The CNN network consists of 8 layers of convolution and a sigmoid classification layer. **Table 3** shows the results of these comparative experiments. On the whole, the performance of NLSE-VNet is the best. However, the CNN network only uses a small number of convolutions to reach a medium segmentation level, therefore CNN has an important role for automatic segmentation.

**Table 3:** Segmentation results of ablation experiments on the BraTS 2020 dataset.

Segmentation	Dice			Sensitivity			Specificity			Hausdorff95		
Model	ET	WT	TC	ET	WT	TC	ET	WT	TC	ET	WT	TC
NL-VNet	0.68	0.83	0.74	0.78	0.90	0.78	0.99	0.99	0.99	44.66	11.16	15.38
SE-VNet	0.72	0.88	0.78	0.75	0.88	0.79	0.99	0.99	0.99	40.76	6.94	12.46
VNet	0.65	0.86	0.76	0.75	0.89	0.84	0.99	0.99	0.99	55.04	9.21	13.28
CNN	0.69	0.86	0.74	0.70	0.87	0.75	0.99	0.99	0.99	52.88	12.07	18.02

**Figure 10:**
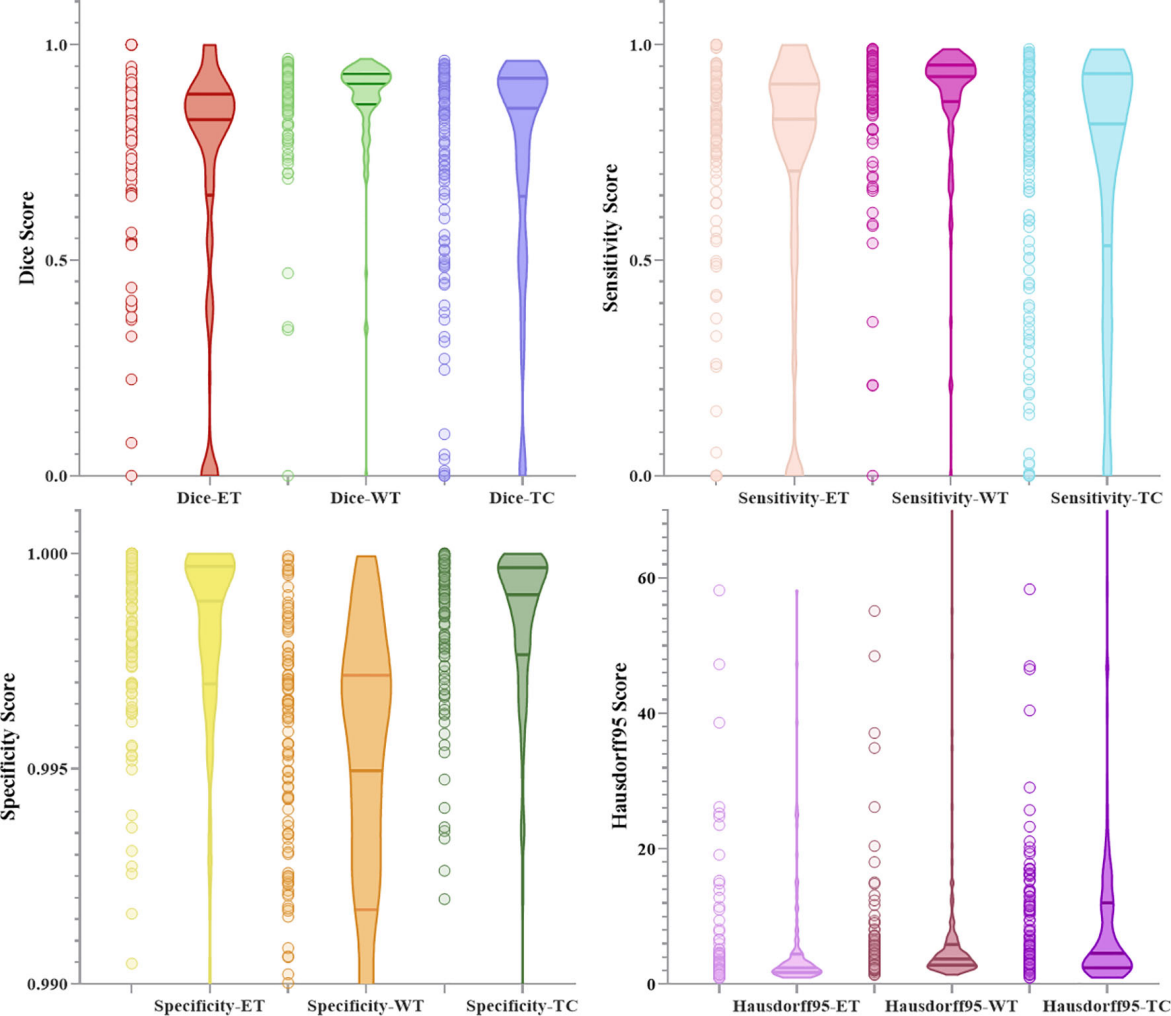
Combination of scatters plot and box plot of the indicators for BraTS 2019.

**Figure 11:**
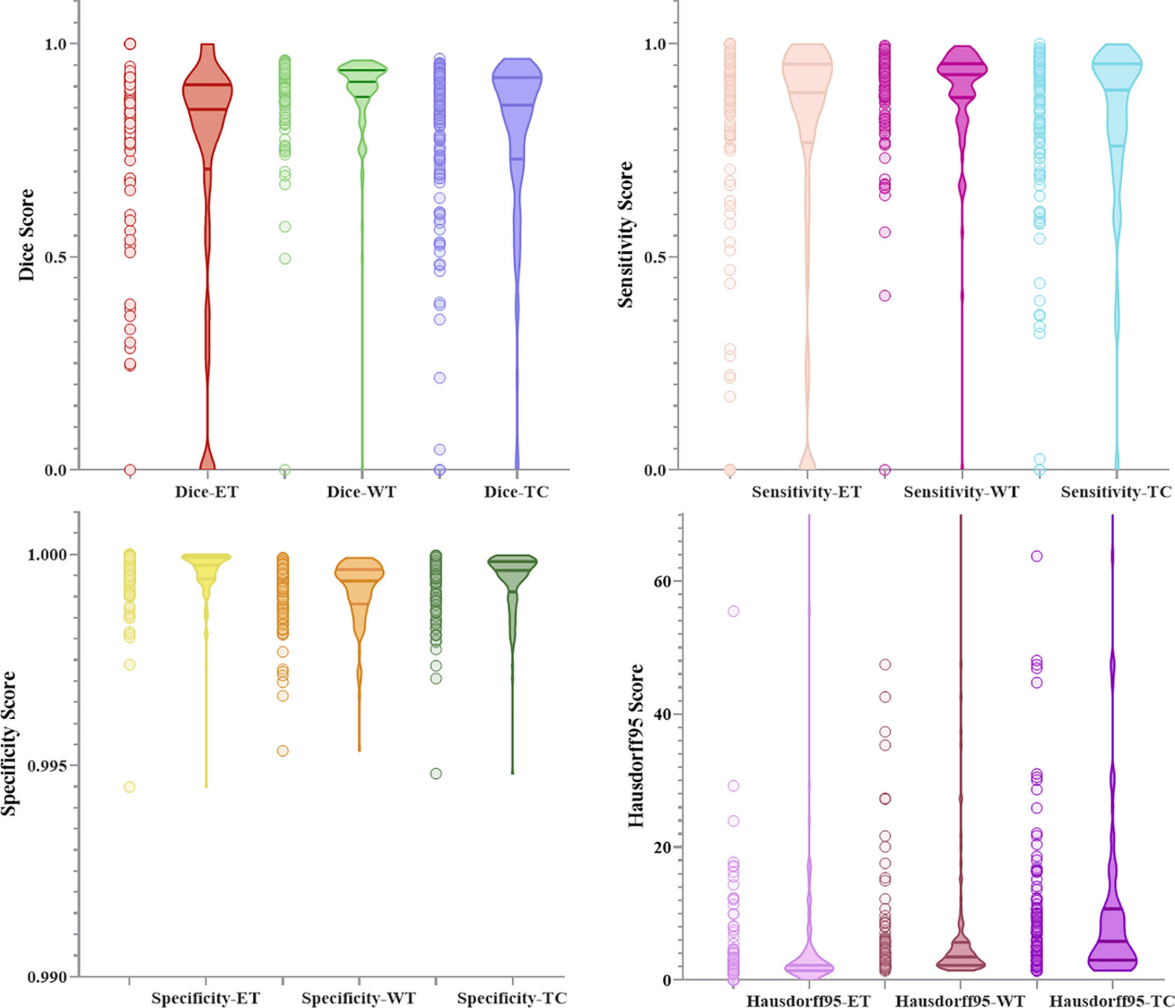
Combination of scatters plot and box plot of the indicators for BraTS 2020.

**Figures 10** and **11** respectively show the combination of violin chart and scatter plot for each evaluation index in the validation set of the NLSE-VNET model on BraTS 2019 and BraTS 2020. It can be seen from the figure that in all samples, the results are relatively concentrated in the higher area, and there are only a few abnormalities, indicating that the model has strong individual case prediction ability. Since sensitivity measures the model’s ability to predict the tumor area, and specificity measures the model’s ability to predict the background, it can be seen that the distribution range of sensitivity is close to the specificity, indicating that the model’s ability to predict the tumor area is similar to the background, effectively alleviating the problem of category imbalance.

**Figure 12:**
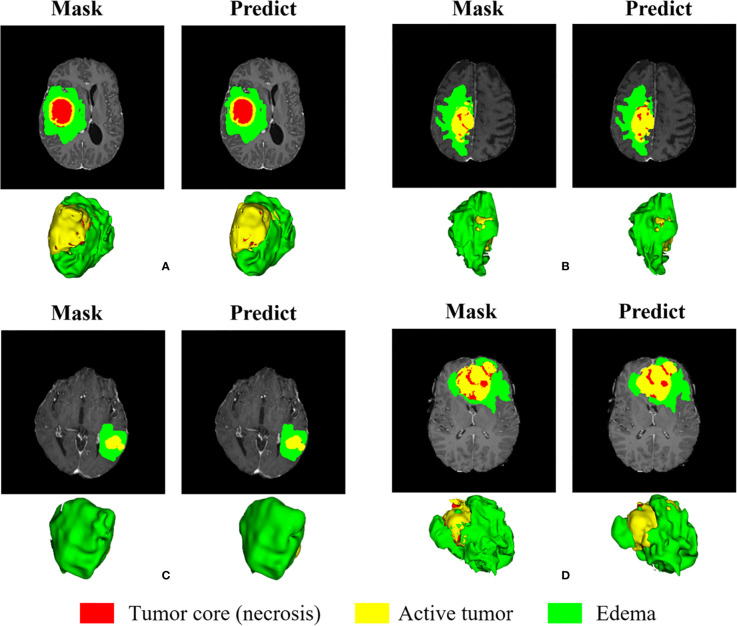
Visual display of brain tumor segmentation results in training set. Red color represents the tumor core (necrosis), yellow color represents the active tumor and green regions are the edema.

We randomly selected four slices in the training set, comparing the experts to the model prediction, while three-dimensional reconstruction of the division results, as shown in **Figure 12**. Among them, red represents the core of the tumor, the combined area of yellow and red represents the enhanced tumor, and the entire segmented area represents the entire tumor. It can be seen that the results of the model are very similar to the standards in overall and detail, providing a more intuitive diagnostic basis for the doctor.

**Figure 13:**
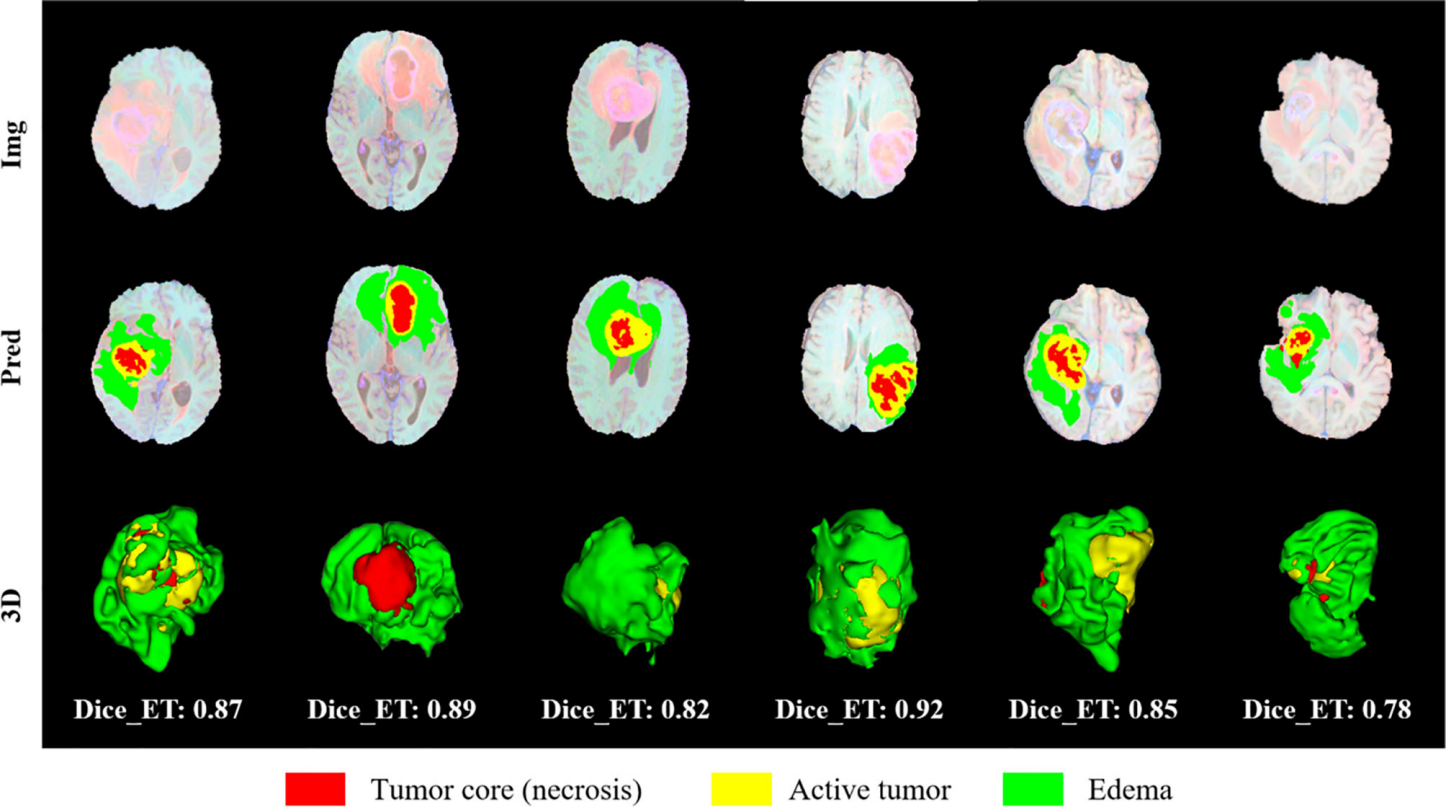
Visual display of brain tumor segmentation results in validation set. Red color represents the tumor core (necrosis), yellow color represents the active tumor and green regions are the edema.

We randomly selected some examples in the validation set to visually present the segmentation results and marked the Dice of the ET area on the graph, as shown in **Figure 13**. We also perform three-dimensional reconstruction of the segmentation results. From these examples, it can be seen that the model is very effective in segmenting tumors of different sizes, shapes, and positions, which also provides an important guarantee for the accuracy of subsequent survival prediction.

### Overall Survival Prediction Results

There are 335 cases in the training set of the BraTS 2019 segmentation task, but the organizer only provided survival labels for 209 cases. BraTS 2020 has 236 cases of lifetime labeling, and due to the error of the segmentation model, the data used in the experiment is 232 cases. The features we use are divided into three categories, clinical factors such as age and tumor grade provided by the organizer, radiological features extracted through the Pyradiomics toolkit, and deep features extracted through CNN. We use these three types of features to obtain the results on the two testsets as shown in **Table 4**.

**Table 4:** The survival prediction result of BraTS 2019 and BraTS 2020.

Dataset	MSE	MAE	RMSE
**BraTS 2019**	96470.98	229.07	306.78
**BraTS 2020**	100053.08	240.05	316.31

At the same time, we also conducted a comparative experiment on the BraTS 2020 dataset. In order to ensure effective comparison, we conducted the same experiment on the mask provided by the organizer and the prediction result of our segmentation model. **Table 5** lists the results obtained by combining different types of features. Among them, “CNN (RF)” represents the use of deep features and random forest models to predict the survival period, and “CNN (DL)” represents the use of deep features and neural networks to predict the survival period.

**Table 5:** Survival prediction results of different types of feature on BraTS 2020.

	Method	MSE	MAE	RMSE
**Mask**	CNN+ Radiology+ Clinical	**87679.85**	**225.59**	**288.84**
	Radiology+ Clinical	88899.18	228.04	292.25
	CNN (RF)	91838.87	235.86	297.16
	CNN(DL)	113472.38	257.71	336.86
**Predict**	CNN+ Radiology+ Clinical	**100053.08**	**240.05**	**316.31**
	Radiology+ Clinical	162904.10	284.54	392.51
	CNN(RF)	153998.46	284.13	378.85
	CNN(DL)	136770.59	269.37	358.92

The bold values provided mean the best performed method.

**Figure 14:**
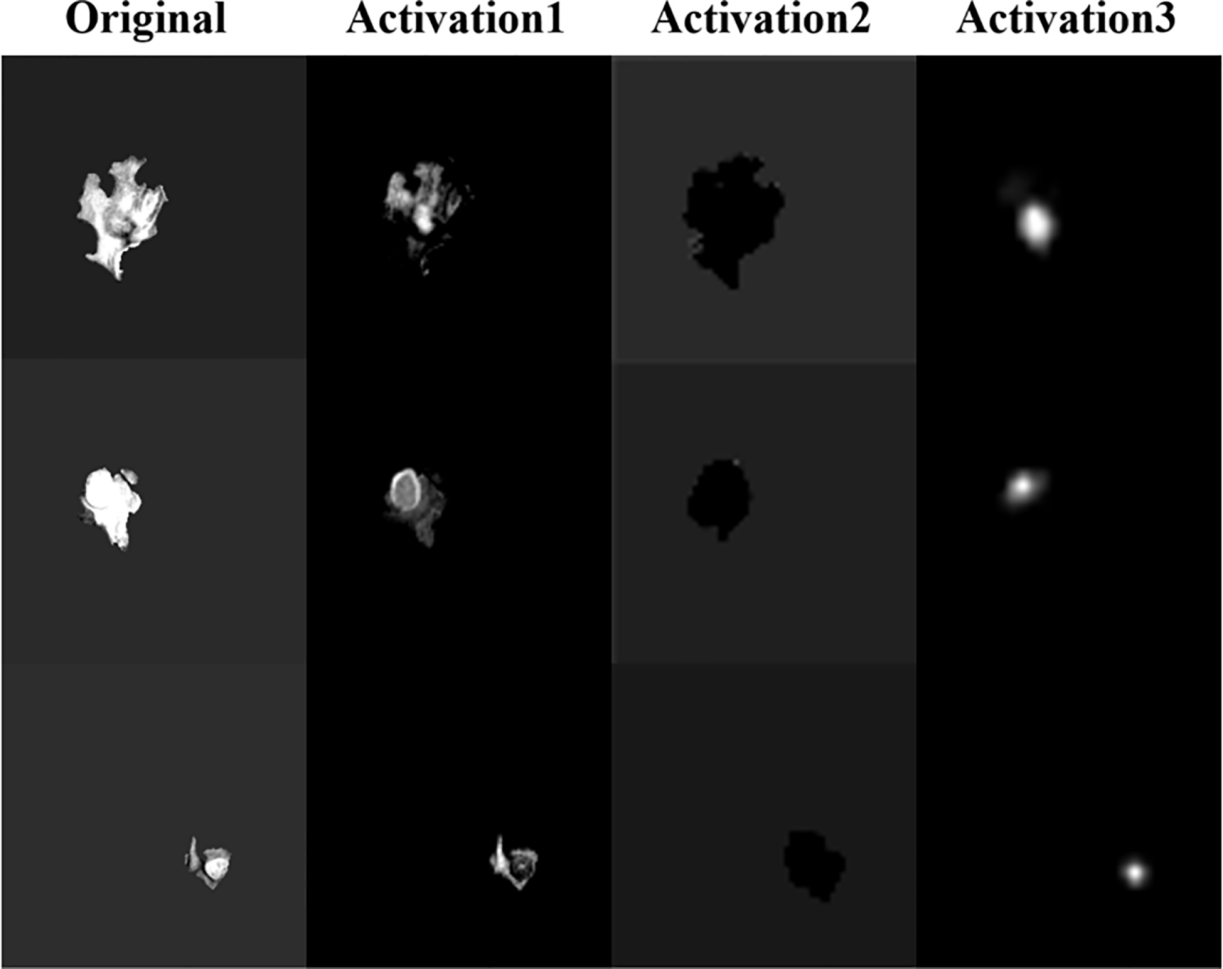
Activation map, the feature map after the ReLU activation layer in the network.

CNN extracting features is usually difficult to explain the biological principles behind it (27). In order to have a deeper understanding of the process of model learning features, we generate activation maps for each activation layer (ReLU) in the network (28), as shown in **Figure 14**. We can observe that after the first activation layer, some features such as texture and intensity are learned, and the second activation layer learns spatial features such as shape and size. It can be seen that the third activation layer focuses attention on In the TC region, we speculate that the features of the TC region are more important for survival prediction than other regions. We can observe that as the number of layers increases, the features extracted by CNN will become more and more abstract.

In order to verify that the radiological features extracted from the original image and the radiological features extracted from the wavelet image can improve the prediction performance, and at the same time prove the importance of the clinical features, we have done comparative experiments, as shown in **Table 6**. “Original” represents the radiological features extracted from the tumor region of the original MR sequence, and “Wavelet” represents the radiological features extracted from the tumor region of the image after wavelet processing. Through the comprehensive comparison in **Table 6**, it is found that the radiological features extracted from the original image are better than those after wavelet in predicting survival. The effect of clinical factors is not as good as we expected. The reason may be that the amount of data is small and the features of clinical factors cannot be reflected.

**Table 6:** Survival prediction results of different radiological features on BraTS 2020.

	Method	MSE	MAE	RMSE
**Mask**	Original+ Clinical	92542.55	233.08	297.83
	Wavelet+ Clinical	89551.18	236.87	296.02
	Original	95207.94	235.46	301.94
	Wavelet	88840.27	236.17	294.93
**Predict**	Original+ Clinical	158230.24	280.82	389.52
	Wavelet+ Clinical	167600.90	292.26	400.93
	Original	157355.79	279.59	388.28
	Wavelet	167466.49	292.26	401.19

**Figure 15:**
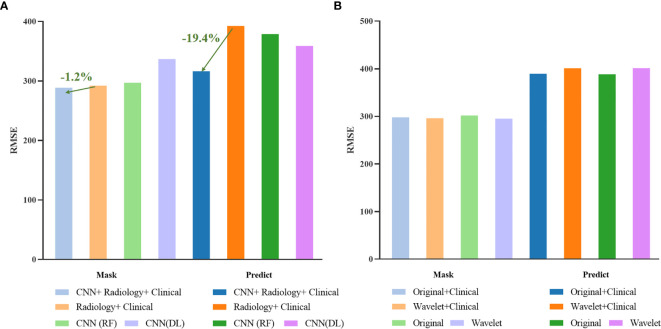
Histogram of survival prediction results.

For intuitive comparison, we show the histogram of the RMSE of each method in **Figure 15**. In **Figure 15** (a), after adding the deep features extracted by CNN, the RMSE has been greatly reduced. Among them, the RMSE of the automatic segmentation results is reduced by 19.4 percentage points, while the survival prediction results using only the deep features are not stable, due to the limitation of data volume, we cannot get more robust results.

**Figure 16:**
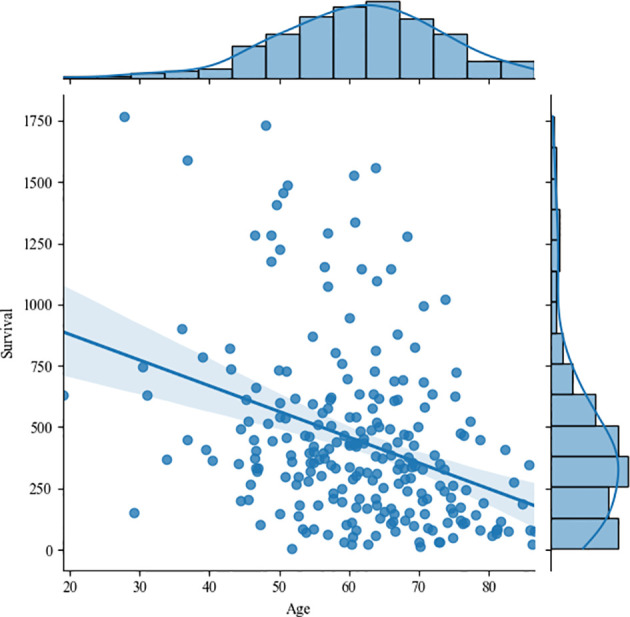
The correlation between age and survival time.

**Figure 16** shows the correlation between age and survival time, and the correlation coefficient is -0.35, that is, there is a weak negative correlation between them. The study of Weninger L. et al. also confirmed our conclusions (29). Since the tumor type of the living time data given by the organizer is HGG, we no longer discuss the correlation between tumor type and survival time. However, it has been proved pathologically that HGG patients with glioma have a poor prognosis, and their survival period is often shorter than that of LGG.

### Comparison

We compare the segmentation results with other methods, as shown in **Table 7**. Kim S. et al. proposes to obtain the initial segmentation probability map with 2D U-Net and then input the MR image and the initial segmentation into the 3D U-Net for segmentation (30). Amian M. et al. proposed a 3D deep segmentation method to divide glioma, including two parallel streamlines having two different resolutions, one convolutional neural network for learning local features, another for the entire image global observation (31). Shi W. et al. adopted a 2D U-Net network segmentation model based on dense cell and feature pyramid unit (32). Agravat R.R. et al. used the three sub-regions of the tumor in the whole convolutional neural network to fuse the segmentation results (33).

**Table 7:** Comparison of the results of the segmentation task.

		Dice			Hausdorff95		
		ET	WT	TC	ET	WT	TC
BraTS 2019	**Proposed**	0.70	**0.87**	0.74	**5.7**	**7.4**	**9.8**
	Kim S. et al. (30)	0.67	0.87	0.76	8.8	14.2	11.7
	Amian M. et al. (31)	**071**	0.86	**0.77**	6.9	8.5	11.6
	Shi W. et al. (32)	0.69	0.87	0.77	5.9	21.2	12.2
	Agravat R.R. et al. (33)	0.60	0.70	0.63	11.7	14.3	17.1
BraTS 2020	**Proposed**	**0.73**	0.88	**0.79**	**36.9**	6.5	**10.0**
	Tarasiewicz T. et al. (34)	0.70	**0.89**	0.75	40.1	**4.6**	10.7
	Mchugh H. et al. (35)	0.71	0.88	0.79	40.6	6.7	10.2
	Zhao C. et al. (36)	0.67	0.86	0.62	47.3	12.6	50.1
	Savadikar C. et al. (37)	0.69	0.82	0.72	36.9	41.5	26.3

The bold values provided mean the best performed method.

At the same time, we listed the results of using the BraTS 2020 dataset. Tarasiewicz T. et al. used Skinny, a lightweight U-Net-based architecture to segment brain tumors, which was originally used to detect skin from color images (34). Mchugh H. et al. believe that two-dimensional segmentation is more advantageous than three-dimensional segmentation. They use 2D density-UNet to segment brain tumors on two-dimensional slices (35). Zhao C. et al. replaced the convolution in the three-dimensional U-Net with two-dimensional multi-view convolution, and learned features in the axial, sagittal, and coronal respectively (36). Savadikar C. et al. used probabilistic U-Net to explore the effect of sampling different segmentation maps, and at the same time explored the effect of changes in the number of attention modules on segmentation quality (37).

We also compare the results of survival prediction in **Table 8**. Kim S. et al. extracted radiomics features, select a small amount of features from random forest retrogenizer to avoid overfitting, and finally predicted the survival time using a random forest regression model (30). Amian M. et al. extracted the spatial features of the entire tumor and sub-organization, using a random forest model to predict the survival time (31). Kofler F. et al. Only uses age this clinical features to predict the survival period, and three orthogonal polynomials and posetric regression models are used (38). Islam M. et al. according to the geometric shape of the tumor, and the position of the new radiology features is combined with clinical features, using XGBooST predictive survival (39).

**Table 8:** Comparison of the results of survival prediction task.

	Team	MSE
**BraTS 2019**	**Proposed**	**96470.98**
	Kim S. et al. (30)	121778.60
	Amian M. et al. (31)	104253.00
	Kofler F. et al. (38)	101877.80
	Islam M. et al. (39)	127478.65
**BraTS 2020**	**Proposed**	**100053.08**
	Soltaninejad M. et al. (40)	109564.00
	Agravat R.R. et al. (41)	116083.48
	Patel J. et al. (42)	152467.00
	Ali M.J. et al. (43)	105079.40

The bold values provided mean the best performed method.

Here are some methods for survival prediction using the BraTS 2020 dataset. Soltaninejad M. et al. used the ratio of tumor volume to brain tissue and the average tumor intensity as features and applied a random forest model to predict survival time (40). Agravat R.R. et al. used a random forest regressor to train the three types of features extracted from shape, volume, and age to predict survival (41). Patel J. extracted 2048 deep features from the segmentation network, used principal component analysis to reduce dimensionality, and trained the Cox risk proportional model for survival prediction (42). Ali M.J. et al. extracted multiple radiological features and image features from the MRI volume, used random forest recursive features to eliminate, and then used random forest regression factors combined with grid search to predict survival (43).

### Discussion

In this section we will discuss the results presented and highlight the shortcomings and solutions in current research.

In the segmentation task, through ablation experiments, we found that the NL module has limited ability to improve segmentation accuracy. It may be that the attention module is not the most suitable model for brain tumor segmentation. We can try to replace the NL module with some other attention modules, such as the SCSE module, which is a variant of the SE module. By focusing on important feature maps or feature channels, it reduces the impact of unimportant features, thereby improving image segmentation results. This module has been applied to brain MR segmentation and achieved excellent results (44). Or Edge Guidance module, which combines edge detection and semantic segmentation, and can use edge information to better supervise and learn semantic segmentation (45). While ignoring a very important concept in medical images, that is, the structure of medical images, we will consider adding structure such as edges and textures when designing segmentation algorithms in the future.

In this article, we design a CNN network to extract the deep features of the image. It contains 4 layers of convolution with a step size of 2 and 3 layers of fully connected neural networks (the last 2 layers of fully connected layers are used to directly predict the survival time). Other network structures were also tried to extract the deep features, and two layers of convolution with a step size of 1 and four layers of convolution with a step size of 1 were added respectively before the fully connected layer. In order to compare which network extracts the best performance of the deep feature, we directly use the fully connected layer to predict the survival time of the deep feature. The results are shown in **Table 9**. Here we only extract deep features from the results of model segmentation. From the results in **Table 9**, it can be seen that the 4-layer convolutional network has the best performance in extracting deep features.

**Table 9:** Survival prediction results of different CNN network structures.

Method	MSE	MAE	RMSE
**CNN with 4 layers of convolution**	**136770.59**	**269.37**	**358.92**
**CNN with 6 layers of convolution**	138065.90	269.83	363.64
**CNN with 8 layers of convolution**	144921.16	301.79	380.69

The bold values provided mean the best performed method.

In the survival prediction task, through the comparative experiments in **Table 3**, we found that only using deep features will make the survival prediction results unstable. The experiment on the mask shows that the neural network results are better than the random forest, while the experiment on the automatic segmentation results has the opposite result, because CNN will show a high degree of variability in different periods. We need more data to fully obtain the robustness of the CNN results. Radiological features have better interpretable advantages and generally more robust results can be obtained. Banerjee et al. designed two new radiological features, extracted from the brain segmentation atlas and spatial habitats and proved their effectiveness (46). We consider introducing these two features in future work to further improve survival prediction performance. In addition, we can use clinical knowledge to classify radiological features more finely, in order to find more suitable feature dimensionality reduction methods, so that feature dimensionality reduction can also have strong interpretability and clinical applicability (47).

## Conclusion

This paper proposes a method for automatically segmenting brain tumors and predicting the survival time of tumor patients, and the performance is verified on the BraTS 2019 and BraTS 2020. First of all, the traditional VNet is improved, and the NLSE-VNet model is proposed. It adds an attention module on the basis of the original V-Net, which can increase a small amount of calculation and greatly improve the segmentation accuracy. Secondly, we used the Pyradiomics toolkit to extract the radiological features of the segmented tumor regions, and the CNN network extracted the deep features (48). Then the factor analysis method is used to reduce the feature dimension, and the clinical features such as age are input into the random forest model to predict the survival period. This research combines promising radiology, machine learning, and deep learning methods, and achieves an average segmentation accuracy of 0.79, and a average RMSE of 311.5 for survival prediction. Experimental results show that this method has reached a relatively prominent level and has good advantages in clinical applications.

## Data Availability Statement

Publicly available datasets were analyzed in this study. This data can be found here: https://www.med.upenn.edu/cbica/brats2020/data.html.

## Ethics Statement

The studies involving human participants were reviewed and approved by Institutional Review Board (IRB), Zhejiang Chinese Medical University. The patients/participants provided their written informed consent to participate in this study.

## Author Contributions

HH, WZ, and XL conceived and designed the study. HH, WZ, YF, JH, SS, and XL contributed to the literature search. HH, WZ, JH, and XL contributed to data analysis and data curation. HH, YF, JH, SS, and XL contributed to data visualization. HH and WZ contributed for software implementation. HH, WZ, YF, JH, and XL contributed to the tables and figures. HH, WZ, YF, SS, and XL contributed to writing of the report. HH, WZ, and XL contribute to review and editing. All authors contributed to the article and approved the submitted version.

## Funding

This work is funded in part by the National Natural Science Foundation of China (Grants No. 62072413, 61602419), in part by the Natural Science Foundation of Zhejiang Province of China (Grant No. LY16F010008), and also supported in part by Medical and Health Science and Technology Plan of Zhejiang Province of China (Grant No. 2019RC224).

## Conflict of Interest

The authors declare that the research was conducted in the absence of any commercial or financial relationships that could be construed as a potential conflict of interest.

## Publisher’s Note

All claims expressed in this article are solely those of the authors and do not necessarily represent those of their affiliated organizations, or those of the publisher, the editors and the reviewers. Any product that may be evaluated in this article, or claim that may be made by its manufacturer, is not guaranteed or endorsed by the publisher.
